# 1006. Association of Development of Pneumonia and Virulence Gene Expression in *Acinetobacter baumannii* Isolated from Clinical Specimens

**DOI:** 10.1093/ofid/ofab466.1200

**Published:** 2021-12-04

**Authors:** Ji Yun Bae, Ina Yun, Kang Il Jun, Chung-Jong Kim, Mi Ae Lee, Hee Jung Choi

**Affiliations:** 1 Ewha Womans University Mokdong Hospital, Ewha Education and Research Center for Infection, Seoul, Seoul-t’ukpyolsi, Republic of Korea; 2 Department of Internal Medicine, Seoul National University College of Medicine, Seoul, Korea, Seoul, Seoul-t’ukpyolsi, Republic of Korea; 3 Ewha Womans University Seoul Hospital, Ewha education and Research Center for Infection, Seoul, Seoul-t’ukpyolsi, Republic of Korea

## Abstract

**Background:**

Not all *Acinetobacter baumannii* isolated from respiratory specimens are true pathogens. Distinguishing between true pathogens and colonizers is important to initiate early treatment and to reduce the unnecessary prescription of antibiotics. To determine the microbiological factors contributing to the development of *A. baumannii* pneumonia, we investigated the association between the expression level of known *A. baumannii* virulence genes such as *ompA* and *hisF* and pneumonia.

**Methods:**

Patients in whose respiratory specimens *A. baumannii* was identified between January 2018 and January 2019 in a tertiary university hospital were recruited into this study. Relevant radiologic findings and more than 5 days of susceptible antibiotic prescription started within 3 days of bacterial isolation were considered as having pneumonia. The absence of radiologic findings of pneumonia until 7 days after the isolation of *A. baumannii* was defined as colonization. The expression of *ompA* and *hisF* was determined with quantitative reverse-transcription polymerase chain reaction. Host factors known to be associated with pneumonia and expression levels of virulent genes were compared between the groups.

**Results:**

Overall, 246 patients in whose respiratory specimens *A. baumannii* was identified were recruited into this study. Among them, 17 and 24 patients were assigned to the pneumonia and colonizer groups, respectively. In the univariable analysis, *ompA*, ICU stay, and mechanical ventilation were significantly associated with pneumonia (p = 0.03, < 0.01, < 0.01 respectively). In the multivariable analysis, mechanical ventilation was significantly associated with pneumonia (OR = 9.75, p = 0.03). *ompA* expression was not significantly associated with pneumonia in the multivariable analysis (OR = 1.12, p = 0.75) (Table 1). *ompA* and *hisF* were significantly associated with the 30-day in-hospital mortality (p = 0.02, < 0.01).

Table 1. Univariable and multivariable analysis of factors related to pneumonia

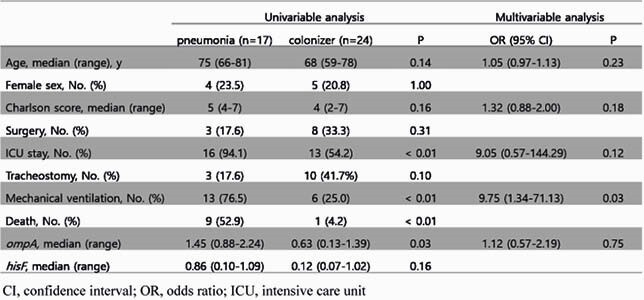

**Conclusion:**

The association between increased *ompA* expression in *A. baumannii* and the development of pneumonia was not statistically significant after adjusting for patient factors. However, the relatively high expression of *ompA* in pneumonia patients and their association with increased mortality suggests the need for larger-scale prospective studies to draw a conclusion.

**Disclosures:**

**All Authors**: No reported disclosures

